# Hibernacula microclimate and declines in overwintering bats during an outbreak of white‐nose syndrome near the northern range limit of infection in North America

**DOI:** 10.1002/ece3.7195

**Published:** 2021-01-20

**Authors:** Karen J. Vanderwolf, Donald F. McAlpine

**Affiliations:** ^1^ Canadian Wildlife Federation Kanata ON Canada; ^2^ New Brunswick Museum Saint John NB Canada; ^3^ Present address: Trent University Peterborough ON Canada

**Keywords:** cave microclimate, fungal disease, microclimate loggers, *Myotis lucifugus*, *Myotis septentrionalis*, *Perimyotis subflavus*, *Pseudogymnoascus destructans*

## Abstract

We document white‐nose syndrome (WNS), a lethal disease of bats caused by the fungus *Pseudogymnoascus destructans* (*Pd*), and hibernacula microclimate in New Brunswick, Canada. Our study area represents a more northern region than is common for hibernacula microclimate investigations, providing insight as to how WNS may impact bats at higher latitudes. To determine the impact of the March 2011 arrival of *Pd* in New Brunswick and the role of hibernacula microclimate on overwintering bat mortality, we surveyed bat numbers at hibernacula twice a year from 2009 to 2015. We also collected data from iButton temperature loggers deployed at all sites and data from HOBO temperature and humidity loggers at three sites. Bat species found in New Brunswick hibernacula include *Myotis lucifugus* (Little Brown Bat) and *M. septentrionalis* (Northern Long‐eared Bat), with small numbers of *Perimyotis subflavus* (Tricolored Bat). All known hibernacula in the province were *Pd*‐positive with WNS‐positive bats by winter 2013. A 99% decrease in the overwintering bat population in New Brunswick was observed between 2011 and 2015. We did not observe *P*. *subflavus* during surveys 2013–2015 and the species appears to be extirpated from these sites. Bats did not appear to choose hibernacula based on winter temperatures, but dark zone (zone where no light penetrates) winter temperatures did not differ among our study sites. Winter dark zone temperatures were warmer and less variable than entrance or above ground temperatures. We observed visible *Pd* growth on hibernating bats in New Brunswick during early winter surveys (November), even though hibernacula temperatures were colder than optimum for in vitro *Pd* growth. This suggests that cold hibernacula temperatures encountered near the apparent northern range limit for *Pd* do not sufficiently slow fungal growth to prevent the onset of WNS and associated bat mortality over the winter.

## Graphical abstract

A 99% decrease in the over‐wintering New Brunswick, Canada, bat population was observed between 2011 and 2015 due to white‐nose syndrome. Cold hibernacula temperatures encountered near the apparent northern range limit for *Pseudogymnoascus destructans* does not sufficiently slow the growth of the fungus to prevent the onset of WNS and associated mortality over the winter.

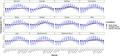

## 1 INTRODUCTION

The emergence and spread of multiple wildlife fungal diseases during recent decades has had devastating consequences for biodiversity (Fisher et al., 2016). When a disease enters a naive host population the initial wave of infection often causes epizootics resulting in mass mortality, which may extirpate local host populations or even cause species extinction (Fisher et al., 2016). Factors not directly associated with interactions between host and pathogen, such as environmental conditions, can considerably influence the manifestation of a disease by acting on either the pathogen or the host (Fisher et al., 2016; Scholthof, 2007).

White‐nose syndrome (WNS), a disease caused by the fungus *Pseudogymnoascus destructans* (*Pd*), is responsible for the deaths of >6.7 million bats in North America (Lorch et al., 2011; US Fish and Wildlife Service, 2019). First reported in 2006 in New York, WNS has spread throughout eastern North America and has also been found in parts of the western USA (Lorch et al., 2016). For unknown reasons, WNS‐associated mortality varies greatly among bat species (Frank et al., 2014; Turner et al., 2011). The WNS‐associated mortality levels of bats in individual hibernacula may be influenced by multiple variables, including distance from the nearest known *Pd*‐positive site, hibernaculum microclimate, initial bat colony size, bat species composition, and latitude (Flory et al., 2012; Langwig et al., 2012; Wilder et al., 2011). However, data reporting WNS‐associated bat declines in North America in relation to the above variables remains scarce in the literature, and no such data exists for Canada (Powers et al., 2015; Turner et al., 2011).

In the USA, *Myotis lucifugus* (Little Brown Bat), *M. septentrionalis* (Northern Long‐eared Bat), and *Perimyotis subflavus* (Tricolored Bat) have suffered up to 100% mortality in multiple caves, with an average of 91%, 98%, and 76% mortality, respectively, in those northeastern hibernacula surveyed (Turner et al., 2011). The arrival of WNS has placed Canadian populations of these species at risk, and all three are now listed as endangered under the Canadian Species‐at‐Risk Act (Environment Canada, 2015). The impact of WNS on Canadian populations of *P. subflavus* may be particularly severe as southeastern Canada represents the northern extent of *P. subflavus* range and this species was considered rare to uncommon in Canada pre‐WNS (Forbes, 2012; Hitchcock, 1965; Mainguy & Derosiers, 2011).

Microclimate is important in hibernaculum selection by bats, among other factors (Boyles et al., 2017), and may also be an important factor influencing WNS‐associated bat mortality rates. Bats in North America often hibernate at sites with high relative humidity and temperatures that range from −10 to 21°C, depending on the bat species and latitude, although the typical range for most cave‐hibernating species is 3.0–10.0°C (Perry, 2013). Cryan et al. (2013) suggested that temperature and relative humidity differences in hibernacula environments could have major effects on which bat species are affected by WNS and infection severity. Indeed, WNS‐associated mortality rates of *M. lucifugus* have been positively associated with hibernacula temperatures in North America (Hayman et al., 2016; Johnson et al., 2014; Langwig et al., 2012, 2016). However, in Europe, Bandouchova et al. (2018) found that *M. myotis* had the highest infection intensity when hibernating at low temperatures. WNS may change the microclimate preferences of bats. Following the arrival of WNS in Pennsylvania, the majority of *M. lucifugus*, *P. subflavus*, and *Eptesicus fuscus* roosted in colder sections of hibernacula compared to pre‐WNS aggregations (Johnson et al., 2016). This behavior may increase energy conservation or affect disease progression (Johnson et al., 2016).

Cave and mine microclimates are influenced by multiple factors, such as local climate, internal hydrology, and internal topology (e.g., length and complexity of passages, number of entrances; Perry, 2013). Generally, cave and mine temperatures reflect mean annual surface temperature of the surrounding area (McClure et al., 2020; Moore & Sullivan, 1997), with the influence of outside weather conditions extending for several hundred meters into sites, depending on topology (Cropley, 1965). Large bodies of water such as subterranean lakes may greatly affect temperatures because of the high specific heat of water (Perry, 2013). Water entering underground sites also affects temperatures; for example warm water entering sites may radiate warmth, even at considerable distances from the entrance (Moore & Sullivan, 1997; Perry, 2013). Most caves and mines have a gradient of increasing relative humidity from the entrance, rising to 100% in the distant reaches of sites (Perry, 2013; Wigley, 1969).

In 2009, anticipating the spread of WNS to Maritime Canada (New Brunswick, Nova Scotia, Prince Edward Island), we initiated surveys of overwintering bats and collected data on species composition, abundance, and microclimate at hibernacula in New Brunswick, Canada (Vanderwolf et al., 2012). Bat populations in New Brunswick hibernacula include *M. lucifugus* and *M. septentrionalis* with small numbers of *P. subflavus* (Vanderwolf et al., 2012). *Eptesicus fuscus* has never been observed in New Brunswick caves or mines, although small numbers overwinter in buildings in the province (McAlpine et al., 2002). Our study area is farther north than most previous hibernacula microclimate studies in North America, and temperatures may be lower, which could affect WNS‐associated mortality rates of bats. Here we present data on the spread of *Pd* among known bat hibernacula in the province following the 2011 arrival of WNS in Maritime Canada, WNS‐associated mortality at individual hibernacula, and the subsequent pattern of decline in overwintering bats in relation to hibernacula microclimate. We also compare hibernacula air temperatures, as measured by different microclimate loggers in the same sites, to assess which physical factors affect hibernacula temperatures.

## 2 METHODS

### 2.1 Bat counts

We monitored six limestone caves, two gypsum caves, and three abandoned manganese mines in southern New Brunswick, Canada, 2009–2015 (site map in Vanderwolf et al., 2012). We also monitored an abandoned copper mine 2011–2015 that was not included in our initial pre‐WNS study (Vanderwolf et al., 2012). These sites (Table 1) included all known bat hibernacula in the province at the time, although additional sites were discovered in 2015. We followed the protocol of the US Fish and Wildlife Service (2012) for minimizing the spread of WNS during all visits to caves, and obtained necessary permits from the New Brunswick Department of Natural Resources and Energy Development.

**TABLE 1:** The number of *Myotis* spp. (*M. lucifugus* and *M. septentrionalis* combined) and *Perimyotis subflavus* in New Brunswick hibernacula ± the standard deviation and the month the count was conducted

	2009	Winter 2010	Winter 2011	Fall 2011	Winter 2012	Fall 2012	Winter 2013
Howes	182 ± 0 Jan	171 ± 4.2 Jan	200.7 ± 6.8 Feb	157 ± 4.2 Nov	221.5 ± 24.7 Feb	128 ± 11.3 Nov	15 ± 0 Apr
	117.3 ± 8 Apr				178 ± 7.1 Mar		
					*118 ± 0 Apr		
Harbells	10 ± 0 Apr	23 ± 1 Jan	31.7 ± 0.6 Feb	14.5 ± 0.7 Nov	33 ± 1.4 Feb	18.5 ± 0.7 Nov	*1 ± 0 Apr
					29 ± 1.4 Mar		
Kitts	ND	24.3 ± 1.2 Feb	15 ± 1 Feb	14.5 ± 0.7 Nov	*19.5 ± 0.7 Feb	4 ± 0 Nov	0 Apr
					6 ± 0 Apr		
Markhamville Mine	>100 Nov	226 ± 7 Feb	151 ± 2.8 Mar	245 ± 5.7 Nov	*268 ± 0 Feb	213 ± 5.7 Dec	12 Apr
					293 ± 9.9 Apr		
					150 ± 1.4 May		
Markhamville Mine 2	ND	ND	ND	1 Nov	*4 Feb	26 Dec	5 Apr
					11 Apr		
					21 May		
Glebe Mine	>100 Nov	159.7 ± 10.3 Feb	155.5 ± 2.1 Mar	192 ± 2.8 Nov	*204.5 ± 7.8 Mar	270 ± 5.7 Dec	22 ± 1.4 Mar
					174 ± 0 Apr		
					113.5 ± 0.7 May		
Underground Lake	336 ± 7.1 Apr	237.3 ± 21.8 Mar	243 ± 4.2 Mar	*203.5 ± 3.5 Dec	56.5 ± 2.1 Mar	18.5 ± 2.1 Dec	13.3 ± 1.5 Mar
					53.3 ± 8 Apr		
					25 ± 1.4 May		
Berryton	ND	934 ± 2.8 Mar	*4,873.5 ± 43.1 Mar	350 ± 1.4 Dec	62.5 ± 0.7 Mar	12 ± 0 Dec	0 Apr
			954.5 ± 40.3 Apr		5 ± 0 Apr		
White	ND	211.8 ± 7.3 Mar	192 ± 17.5 Mar	*218.5 ± 0.7 Dec	114.5 ± 10.6 Feb	23 ± 1.4 Dec	9 ± 1 Apr
					45.5 ± 0.7 Apr		
					31.3 ± 0.6 May		
Dorchester Mine	ND	ND	ND	*140 ± 17 Dec	34 ± 4.2 Mar	5 ± 0 Dec	0 Mar
					1 ± 0 Apr		
Dallings	3 ± 0 Nov	0 Feb, Nov	0 Feb	3 ± 0 Dec	*1 ± 0 Mar	6 ± 0 Nov	2 ± 0 Apr
Chantals	0 Nov	0 Mar, Nov	0 Mar	0 Dec	ND	ND	ND

	Fall 2013	Winter 2014	Fall 2014	Winter 2015
Howes	7 ± 0 Dec	5 ± 0 Jan; 6 ± 0 Apr	3 ± 0 Dec	4 ± 0 Apr
Harbells	0 Dec	0 Apr	0 Dec	0 Apr
Kitts	0 Dec	0 Apr	0 Dec	0 Apr
Markhamville Mine	9 ± 0 Dec	2 ± 0 Apr	4 ± 0 Dec	3 ± 0 Apr
Markhamville Mine 2	0 Dec	ND	0 Dec	0 Apr
Glebe Mine	1 ± 0 Dec	0 Apr	1 ± 0 Nov	0 Apr
Underground Lake	3 ± 0 Dec	2 ± 0 Apr	0 Nov	0 Apr
Berryton	1 ± 0 Dec	0 Apr	2 ± 0 Nov	0 Apr
White	15 ± 0 Dec	12 ± 0 Apr	11 ± 0 Nov	5 ± 0 Apr
Dorchester Mine	3 ± 0 Dec	2 ± 0 Mar	1 ± 0 Dec	1 ± 0 Mar
Dallings	0 Dec	0 Apr	0 Nov	0 Apr
Chantals	ND	0 May	ND	0 May

Note

The numbers include live bats only. Counts in bold with a * indicate the first time visible *Pd* growth was observed on roosting bats in each hibernaculum.

Abbreviation: ND, no data.

We counted hibernating bats twice annually (November–December and March–April) as in Vanderwolf et al. (2012), ~6–8 weeks after bats had entered hibernacula for the winter and ~3–6 weeks before estimated exit from each site for the summer. We did this to determine how many bats were present at the start of hibernation and how many survived to the end of hibernation in each site. Typically, each count was undertaken by two individuals. Low visitation rates (e.g., 1–3 visits per winter) are reported to have little detrimental effect as hibernating bats tolerate several forms of natural disturbance, such as sound and predator activity (Boyles, 2017; Kilpatrick et al., 2020). When present, we collected and identified bat carcasses to species and sex. To reduce disturbance, live *Myotis* were not identified to species or sex. However, we identified *Myotis* to species and determined sex for ten live bats per hibernaculum in 2010 (*n* = 8 hibernacula) and species only in 2012 (*n* = 5 hibernacula) when handling bats was required for other research (Vanderwolf et al., 2013, 2016). Hibernating *P*. *subflavus* are morphologically distinct and could be identified without removing bats from cave walls, therefore this species was identified throughout the study. To minimize disturbance to hibernating bats we assessed live bats in the field for the presence of characteristic *Pd* fungal growth by visual inspection of exposed skin surfaces while bats where roosting (i.e., we did not remove bats from cave walls for inspection). This method underestimates the number of bats with WNS, as we could only examine the exposed skin of accessible roosting bats (some bats were roosting too high to be assessed). As well, lack of visible *Pd* growth does not equate to the absence of WNS (Janicki et al., 2015). One bat per hibernaculum was removed for confirmation of WNS by histology and sequencing at the Canadian Cooperative Wildlife Health Center on Prince Edward Island after our first observation of visible *Pd* growth at a site.

### 2.2 Hibernacula microclimate

We placed air temperature logger iButtons (model DS1921G, ±1°C, Maxim Integrated Products, Inc.) in caves and mines December 2011 and retrieved them August 2017. We set iButtons to record air temperature twice daily (02:30 and 14:30 hr) to capture daily temperature extremes and increase longevity of the devices. We deployed three iButtons at each site: We placed one 1–2 m from the ground on a wall ledge near the ceiling in the twilight zone (i.e., the entrance area where some light penetrates 7 ± 5 m into the cave), a second under similar circumstances in the dark zone (zone where no light penetrates), and attached a third to a tree at chest height 50–200 m from each hibernaculum entrance. We placed iButtons in the dark zone in passage or chamber areas where bats roosted (if bats were present) 45 ± 26 m *SD* (range 16–200 m) from the entrance, with placement depending on hibernaculum length and roosting location of hibernating bats. Dark zone iButtons were placed in areas with the largest concentration of bats, which was generally not the deepest part of each hibernaculum (total lengths ranged from 74 to 515 m). To avoid loss inside caves, we placed iButtons in perforated (to facilitate airflow) translucent plastic boxes. iButtons outside caves were placed in plastic camouflaged boxes (painted brown to deter theft) with a hole drilled in the bottom to allow water drainage. Due to iButton failures, we placed two iButtons in each box starting in 2013. When available we used the mean value of paired iButtons for analysis.

We deployed temperature/relative humidity loggers (HOBO model U23‐001; ±0.45°C, ±2.5% from 10% to 90% RH and ± 5% at >90% RH, Onset Computer Corporation, Bourne, MA) at three sites in conjunction with iButtons: White Cave, Markhamville Mine, and Berryton Cave. We deployed HOBOs April 2014 and retrieved them August 2017. We placed three HOBOs at each site near existing iButtons (twilight zone/entrance, dark zone, and outside the hibernaculum) and programmed them to record air temperature and relative humidity twice daily (02:30 and 14:30 hr). We tied HOBOs outside hibernacula at chest height to the same tree as camouflaged iButton boxes, with the HOBO placed just above the iButtons and the sensor pointing toward the ground. We wrapped HOBOs in a tube of black foam pipe insulation for protection and camouflage, while ensuring that the sensor was open to the air at the bottom of the tube. Loggers tied to trees were below the forest canopy, which probably modulated local extremes in temperature and humidity. We downloaded data from iButtons and HOBOs twice a year during bat counts and replaced malfunctioning iButtons. The relative humidity sensor of the HOBO logger deployed in the dark zone of White Cave failed September 2016, so no data were available after that date.

During visits March 2012–May 2015 we also measured air temperature and relative humidity with a Kestrel 3000 Handheld weather meter (MPN# 0830, ±0.04°C, ±1% RH). Kestrel measurements took some time to stabilize, particularly for relative humidity, so the device was temporarily attached to a tree adjacent to the hibernaculum entrance for measurements above ground and positioned on the hibernacula floor for measurements inside passages while we performed bat counts.

### 2.3 Statistical analysis

We converted relative humidity to water vapor pressure because relative humidity is less informative and potentially misleading (Kurta, 2014). We calculated equilibrium vapor pressure using temperature data and the quadratic formula of Tabata (1973), and then determined actual vapor pressure (hPa) by multiplying the saturation vapor pressure by the relative humidity as recorded by HOBOs. We incorporated previous iButton temperature data collected from the same sites October 2009 to October 2010 (Vanderwolf et al., 2012) in the analysis. We conducted all analyses in R (R Core Team, 2020), and generated plots using ggplot2 (Wickham, 2016).

To determine if there were temporal or geographic patterns in temperature and humidity data, we ran two separate linear models [package lme4; (Bates et al., 2015)] with location (dark zone, twilight zone, outside), site, year (as a categorical variable), month, and two interaction terms (month:location and year:location) as explanatory variables: one model with iButton temperatures as the dependent variable and the second model with actual water vapor pressure as calculated from HOBO measurements. After determining that hibernacula temperatures varied by site, we ran two separate linear mixed effects models [package lme4; (Bates et al., 2015)] using iButton temperature data to determine which site characteristics affected hibernacula temperatures: one model for the twilight zone and the second for the dark zone with hibernacula length (distance from entrance to site of logger placement, rather than total hibernaculum length) and presence of water (standing, flowing, or no water) as fixed effects, and year and month as random effects. We compared temperature data taken by iButtons and HOBOs placed in the same locations within the same sites and paired iButtons (in the same box) with a Wilcox test and a paired Wilcox test, respectively. We compared air temperature (as measured by iButtons) to water temperature data collected during a previous study (Vanderwolf et al., 2017) with a Wilcox test.

To determine which variables affected the number of bats in each cave, we ran a linear model with hibernaculum passage length, presence of water, year, mean dark zone temperature in the winter (November–April), winter standard deviation of dark zone temperature, maximum winter dark zone temperature, and minimum winter dark zone temperature as explanatory variables. The April bat count was used as the number of bats at each site for each winter, and the temperature variables were calculated from iButton measurements. We used the function AICtab (package bbmle) (Bolker & Team, 2017) to compare model Akaike information criteria (AIC) values. We considered a *p* < .05 significant.

## 3 RESULTS

### 3.1 Bat counts

Berryton Cave, the hibernaculum with the greatest number of bats in New Brunswick, was the first WNS‐positive site in the province and Maritime Canada (March 2011). Bats in the three hibernacula closest to Berryton Cave were WNS‐positive by fall 2011, while bats in the two hibernacula farthest from Berryton Cave, but closest to WNS‐positive locations in the adjacent USA, were confirmed last. All known hibernacula in the province were *Pd*‐positive with WNS‐positive bats by winter 2013, 2 years after WNS first appeared in the Maritimes. Prior to the arrival of WNS in New Brunswick, the closest known *Pd*‐positive site was in western Maine, approximately 500 km from the nearest known New Brunswick hibernacula (Howes Cave and Harbells Cave) and 650 km from Berryton Cave (located in the southeast of the province).

Counts of overwintering bat populations in ten New Brunswick hibernacula went from a high of 7,076 bats in winter 2011 to 13 individuals in winter 2015 (Table 1). White Cave had higher numbers of bats fall 2013–winter 2015 compared to other sites. The number of bats counted in two sites spiked when WNS was first observed (Berryton Cave March 2011 and Glebe Mine December 2012), but not in the other sites. Pre‐WNS, bat counts peaked midwinter while post‐WNS counts were highest in the fall. The first detection of visible *Pd* growth on bats in each site generally coincided with population decreases (Table 1). Notable exceptions were Howes Cave, Markhamville Mine, and Glebe Mine, which each had a delay of 1 year between the first detection of visible *Pd* growth on bats and a decrease in hibernating bat populations.

The number of bats with visible *Pd* growth increased over the hibernation period (Table 2), with more luxuriant fungal growth observed later in the winter. The earliest date we observed visible *Pd* growth in any year was November 18; this is also the earliest date we carried out surveys over all years. Generally, bats roosting near the entrances of hibernacula were more likely to have visible *Pd* growth than those roosting deeper in hibernacula. In Berryton Cave in March 2011 40% of bats near the entrance (*n* = 257 bats) had visible *Pd* growth compared to 29% of bats roosting deep in the cave (*n* = 571 bats). Fungal growth on bats was less common and less luxuriant in the years after WNS was first observed in the province (Table 2).

**TABLE 2:** The number of freshly dead *Myotis* spp. and *Perimyotis subflavus* seen in New Brunswick hibernacula and the percentage of live bats with visible WNS

	Winter 2011	Fall 2011	Early Winter 2012	Mid‐Winter 2012	Late Winter 2012	Fall 2012	Winter 2013	Fall 2013	Winter 2014	Fall 2014	Winter 2015
Howes	0, 0%	0, 0%	0, 0%	0, 13%	0, 39%	0, 1.1%	0, 93.3%	0, 71.4%	0, 40% Jan; 0, 83.3% Apr	0, 33.3%	0, 75%
Harbells	0, 0%	0, 0%	0, 0%	0, 0%	ND	0, 0%	0, 100%	0, n/a	0, n/a	0, n/a	0, n/a
Kitts	0, 0%	0, 0%	1, 25%	0, 50%	ND	0, 25%	0, n/a	0, n/a	0, n/a	0, n/a	0, n/a
Markhamville Mine	0, 0%	0, 0%	3, 25%	0, 43.9%	0, 65%	0, 19.6%	1, 58%	0, 33.3%	0, 0%	0, 0%	0, n/a
Markhamville Mine 2	ND	ND	ND	0, 71.4%	2, 21.2%	1, 38.5%	5, 40%	0, 0%	0, 0%	0, 0%	0, 0%
Glebe Mine	0, 0%	0, 0%	0, 27.6%	0, 56%	0, 53%	0, 27.6%	18, 9.1%	0, 0%	0, n/a	0, 100%	0, n/a
Underground Lake	0, 0%	0, 60%	16, 54.2%	4, 34.3%	1, 50%	0, 0%	0, 100%	0, ?	0, 100%	0, n/a	0, n/a
Berryton	1,214, 34.3% Mar; 548, 46% Apr	0, 9.3%	28, 41.6%	17, 100%	ND	0, 33%	0, n/a	0, 0%	0, n/a	0, 0%	0, n/a
White	0, 0%	1, 29.4%	2, 70.4%	1, 93.5%	1, 83%	0, 0%	0, 0%	0, 100%	0, 0%	0, 0%	0, 100%
Dorchester Mine	ND	5, 25.7%	26, 73.5%	11, 100%	ND	0, 60%	2, n/a	0, 0%	0, 50%	0, 0%	1, 100%
Dallings	0, 0%	0, 0%	ND	2, 100%	ND	0, 0%	6, 0%	0, n/a	0, n/a	0, n/a	0, n/a

Note

In some hibernacula, bats were roosting in areas where they could not be adequately assessed to determine visible WNS (particularly White Cave and Underground Lake Cave). Only bats that could be adequately assessed were used to calculate percentages.

Abbreviations: ?, live bats present but could not be assessed; n/a, no live bats present; ND, no data.

More bats roosted near the entrance of Glebe Mine and Berryton Cave during WNS‐associated mortality events compared to pre‐WNS. Fewer than five bats were observed near each entrance at Dorchester Mine, Kitts Cave, and Dallings Cave after the initial introduction of *Pd*. Bats were not observed near entrances pre‐WNS or in the years following WNS‐associated mortality events at these sites, and were never observed near entrances in White Cave, Underground Lake Cave, Markhamville Mine, Howes Cave, or Harbells Cave.

Although bat numbers declined at all sites, large numbers of bat carcasses were only observed in Berryton Cave (Table 2). We suggest that bats emerged from the other hibernacula and died on the landscape due to exposure/starvation/predation, as has been noted elsewhere (Turner et al., 2011). We observed bats exiting hibernacula in the daytime during winter at Berryton Cave, Glebe Mine, and Underground Lake Cave. In March 2011, we counted 607 ± 37 bat carcasses on the floor of Berryton Cave with an additional 26 carcasses outside the entrance. It is likely that an unknown number of bats had exited the cave prior to our survey. In April 2011, we counted 505 ± 6 fresh bat carcasses on the floor of Berryton Cave, but none outside. During counts in Berryton Cave in March and April 2011, bats hanging from the cave walls and ceiling were not assessed as live or dead, thus underestimating the number of bat carcasses. In all subsequent counts, we distinguished live bats from dead bats (Tables 1 and 2). Most bat carcasses in Berryton Cave winter 2011 were predated by raccoons (McAlpine et al., 2011) or removed for research purposes (*n* = 588). We archived carcasses in the New Brunswick Museum (NBM# 012497‐012499, 012780‐012798, 012933‐012945, 014238, 014273‐014296). Nevertheless, many bat skeletons were still present on the floor and walls of Berryton Cave summer 2017, particularly near the entrance. Other hibernacula had zero to <5 bat skeletons remaining as of winter 2015.

Identification of carcasses, as well as live bats, revealed that most known New Brunswick hibernacula were dominated by *M. lucifugus*, particularly males, although some hibernacula appeared to be dominated by *M. septentrionalis* (Table 2). *Myotis septentrionalis* appeared to be more common in Dorchester Mine pre‐WNS compared to post‐WNS as W.S. Stallworthy and students of Mount Allison University banded 686 *Myotis* spp. (roughly equal numbers of *M. lucifugus* and *M. septentrionalis*) at the mine 1956–1963 (unpublished report, New Brunswick Museum file: *Myotis* sp. 1956–NB). *Perimyotis subflavus* were less common at our study sites than *Myotis* spp. and concentrated in Glebe Mine and Markhamville Mine (Tables 3 and 4). Pre‐WNS (2009–2011), we observed 12–14 each fall and 16–21 *P. subflavus* each winter. The population decreased after the arrival of WNS, and we did not observe any *P. subflavus* during surveys December 2013–April 2015. *Perimyotis subflavus* hibernated in the same chambers as *Myotis* spp., but roosted singly and low on the walls, generally 1–2 m from the hibernaculum floor.

**TABLE 3:** Total number of each bat species and sex identified during winter in New Brunswick hibernacula 2009–2015

	Harbells Cave	Howes Cave	Kitts Cave	Mark Mine	Glebe Mine	Lake Cave	Berryton Cave	White Cave	Dallings Cave	Dorchester Mine	Chantal Cave	Total
*Myotis lucifugus*	4	13	0	17	8	3	548	21	10	17	0	618
*M. septentrionalis*	16	9	11	4	5	9	44	2	7	8	0	113
*Perimyotis subflavus*	4	0	0	45	39	2	4	4	2	0	0	100
Male	7	9	3	10	7	7	370	9	4	9	0	418
Female	2	1	3	2	5	5	231	3	1	15	0	251
Max Bats	33	222	24	293	270	336	4,874	218	6	140	0	
Mean Temp	5.8 ± 2.2	5.9 ± 1.8	4.9 ± 2.4	5.0 ± 0.6	5.8 ± 1.9	3.3 ± 1.0	4.6 ± 0.8	5.1 ± 1.1	4.5 ± 2.2	6.6 ± 0.3	1.5 ± 2.4	
Max Temp	12.1	9.6	10.4	8.4	9.7	8.1	6.6	7.66	10.1	7.1	10.1	
Min Temp	2.6	3.1	1.5	3.1	3.1	1.1	2.3	3.1	−1.0	6.1	−4.4	

Note

Max bats indicates the maximum number of bats counted, but not identified to species, in each cave during one visit in the period 2009–2015. Mean winter temperature (°C) ± standard deviation and maximum and minimum winter temperatures recorded in the dark zone with iButtons 2009–2017 are listed. Winter is defined as November–April. Mark and Lake are short for Markhamville Mine and Underground Lake Cave, respectively.

**TABLE 4:** The number of live *Perimyotis subflavus* seen in New Brunswick hibernacula with the month the count was conducted and the percentage of bats with visible fungal growth on exposed skin

	Winter 1977	Winter 2010	Fall 2011	Winter 2012	Fall 2012	Winter 2013	Fall 2013	Winter 2014	Fall 2014	Winter 2015
Howes	ND	0 Jan	0 Nov	0 Feb, 0 Mar, 0 Apr	0 Nov	0 Apr	0 Dec	0 Jan, 0 Apr	0 Dec	0 Apr
Harbells	ND	1 Jan	1 Nov	1 Feb, 1 Mar	1 Nov	0 Apr	0 Dec	0 Apr	0 Dec	0 Apr
Kitts	ND	0 Feb	0 Nov	0 Feb, 0 Apr	0 Nov	0 Apr	0 Dec	0 Apr	0 Dec	0 Apr
Markhamville Mine	ND	9 Feb	6 Nov	7 Feb, 6 Apr, 5 May	7 Dec 42.9%	3 Apr 100%	2 Dec	0 Apr	0 Dec	0 Apr
Markhamville Mine 2	ND	ND	ND	1 May	0 Dec	0 Apr	0 Dec	0 Apr	0 Dec	0 Apr
Glebe Mine	1 Oct	5 Feb	7 Nov	7 Mar, 7 Apr, 5 May	4 Dec	2 Mar 50%	1 Dec	0 Apr	0 Nov	0 Apr
Underground Lake	1 May	1 Mar	0 Dec	0 Mar, 0 Apr, 0 May	0 Dec	0 Mar	0 Dec	0 Apr	0 Nov	0 Apr
Berryton	2 Oct	2 Mar	0 Dec	0 Mar, 0 Apr	0 Dec	0 Apr	0 Dec	0 Apr	0 Nov	0 Apr
White	ND	3 Mar	0 Dec	1 Feb, 0 Apr, 0 May	0 Dec	0 Apr	0 Dec	0 Apr	0 Nov	0 Apr
Dorchester Mine	ND	ND	0 Dec	0 Mar, 0 Apr	0 Dec	0 Mar	0 Dec	0 Mar	0 Dec	0 Mar
Dallings	ND	0 Feb	0 Dec	0 Mar	0 Nov	1 Apr + 1 dead	0 Dec	0 Apr	0 Nov	0 Apr

Note

If no fungal growth was seen a percentage is not given. An additional *P. subflavus* was observed in Dallings Cave November 2009 (Vanderwolf et al., 2012), one in Kitts Cave Feb 1976 (McAlpine, 1976), and one near Greenhead Cave Aug 1976 (Broders et al., 2001). *Perimyotis subflavus* were not differentiated from *Myotis* spp. during bat surveys in 2009 or winter 2011, although a dead *P. subflavus* was collected from Berryton Cave March 2011. ND = no data. Data from 1977 taken from (Broders et al., 2001). Data from 2010 taken from (Vanderwolf et al., 2012).

Pre‐WNS, *Myotis* spp. were observed roosting singly (i.e., no clusters) in Dallings Cave, Kitts Cave, and Markhamville Mine 2. Small clusters (<20 individuals) were observed in Dorchester Mine, Harbells Cave, Howes Cave, and White Cave, although most bats roosted singly. Large clusters (<100 individuals but >20) were observed in Berryton Cave, Glebe Mine, Underground Lake Cave, and Markhamville Mine. No clusters were observed at any site in the years following WNS‐associated mortality events.

### 3.2 Cave microclimate

The number of microclimate readings taken at each site varied depending on how many loggers malfunctioned (Table 5). Overall, we collected 138,061 iButton air temperature readings (counting paired iButtons in the same box as a single measurement) and 21,842 HOBO air temperature readings.

**TABLE 5:** Number of readings taken with two types of microclimate loggers. This includes data from Vanderwolf et al. (2012)

Site	No. of iButton readings	No. of HOBO readings		Presence of water
		Temp	RH	
Berryton Cave	13,232	7,278	7,277	Small, seasonal, stationary pools
Chantal Cave	8,931^a^	NA	NA	Flowing stream throughout
Dallings Cave	13,447	NA	NA	Flowing stream throughout exiting the main entrance
Dorchester Mine	12,064	NA	NA	Flowing stream exiting the entrance and stationary pools
Glebe Mine	9,576	NA	NA	Small stationary pools and small seasonal stream at the end of the mine
Harbells Cave	11,424	NA	NA	Flowing stream throughout flowing into the cave
Howes Cave	11,610	NA	NA	One small stationary pool at the end of the cave
Kitts Cave	11,367	NA	NA	Flowing stream throughout the cave and exiting the entrance
Underground Lake Cave	13,952	NA	NA	Large stationary pool
Markhamville Mine 2	7,158^b^	NA	NA	Small stationary pools
Markhamville Mine	12,135	7,292	7,291	Stationary pools at the end of the mine
White Cave	13,165	7,272	6,580^c^	Very small flowing stream at the end of the cave
Total	138,061	21,842	21,148	

Note

HOBO readings include both temperature and relative humidity. Numbers indicate the sum total of readings taken by loggers deployed outside, in the twilight zone, and the dark zone at each site. Only the mean value is counted for paired iButtons. NA = no HOBOs deployed at the site. The type of water body in each site is described.

^a^ iButtons removed from Chantal Cave May 2015.

^b^ iButtons first deployed in Markhamville Mine 2 December 2014.

^c^ HOBO RH sensor in the dark zone of White Cave malfunctioned so RH data from September 2016–August 2017 were discarded.

**FIGURE 1:**
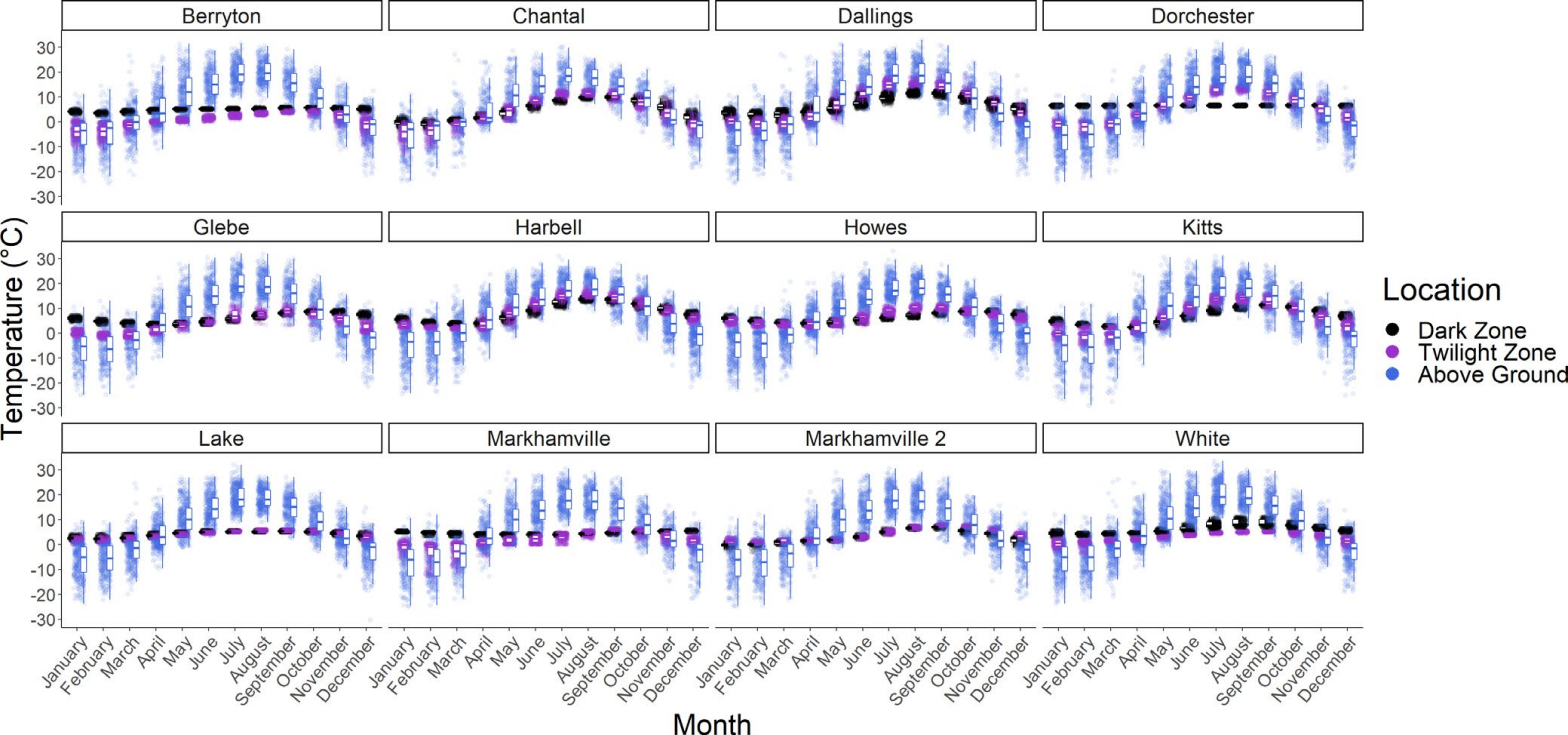
Temperatures as measured by iButton loggers in New Brunswick, Canada, caves and mines 2009–2017. “Above Ground” refers to 50–200 m distant from the cave or mine entrance

Air temperatures at sites, as measured with iButtons, varied among months (*F*_11_ = 18,374.7, *p* < .001), locations within sites (*F*_2_ = 4,453.7, *p* < .001), sites (*F*_11_ = 1,417.3, *p* < .001), and years (*F*_8_ = 318.7, *p* < .001). There were also interactions between location and month (*F*_22_ = 5,360.3, *p* < .001), location and site (*F*_22_ = 455.8, *p* < .001), and location and year (*F*_14_ = 31.6, *p* < .001) as dark zone temperatures did not significantly vary with month, site, and year, unlike above ground and twilight zone temperatures. Overall, twilight zone temperatures were lower (estimate = 0.67°C, *p* < .001) than dark zone temperatures (estimate = 1.95°C, *p* < .001), while above ground temperatures were higher (estimate = 2.95°C, *p* < .001), although these patterns differed seasonally. Above ground temperatures were more variable than either dark or twilight zone temperatures (Figure 1, Table 6). In most sites, dark zone temperatures were warmer than twilight zone and above ground temperatures in the winter, and colder in the summer.

**TABLE 6:** Mean temperature ± standard deviation (°C) measured by iButton loggers for 12 caves/mines in New Brunswick 2009–2017

Location	Winter	Summer	Year‐Round
Dark Zone	4.73 ± 2.18	7.00 ± 2.57	5.87 ± 2.64
Twilight Zone	1.48 ± 3.26	7.41 ± 4.22	4.40 ± 4.79
Outside	−1.34 ± 7.32	14.77 ± 6.29	6.66 ± 10.56

Note

Winter = November–April; Summer = May–October. See text for location definitions.

**FIGURE 2:**
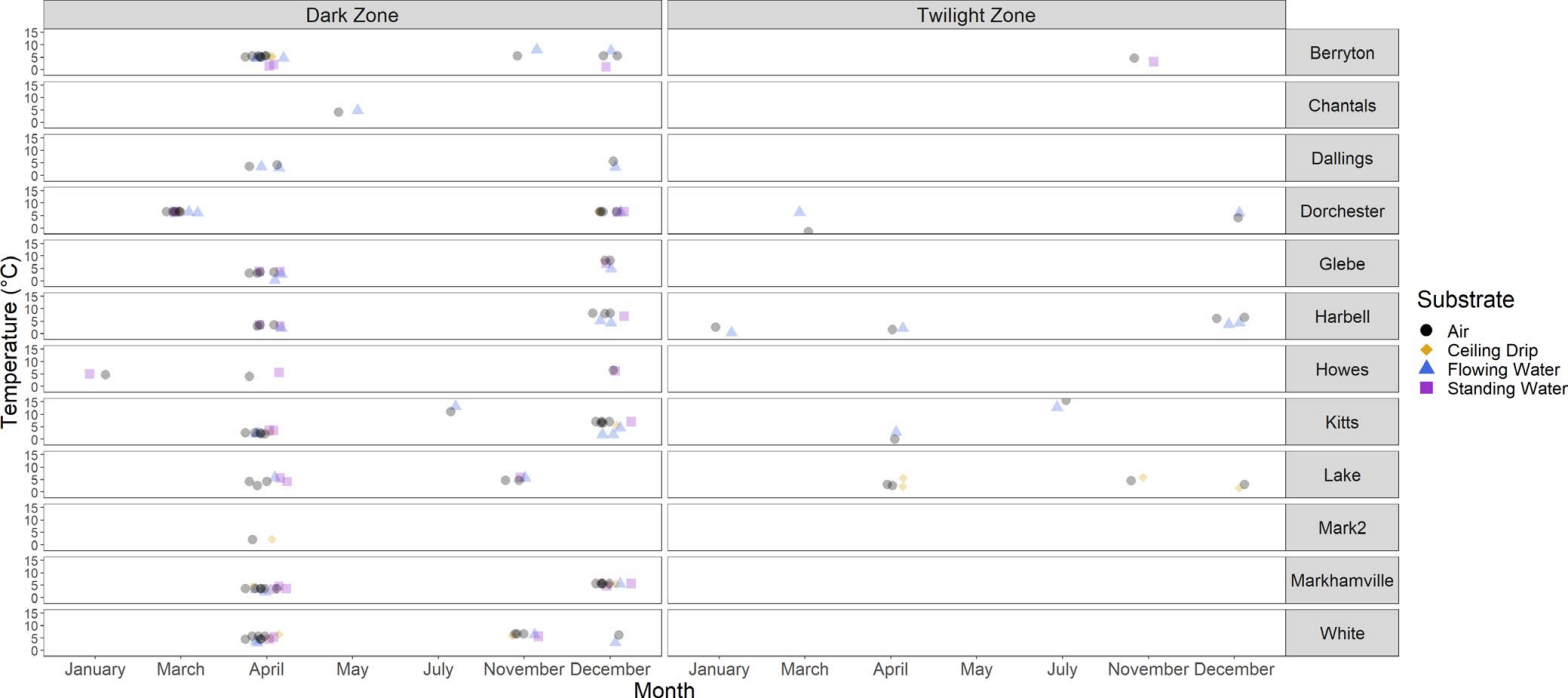
Air temperatures of caves in New Brunswick measured by iButtons on the same day and time that water temperatures were measured. Water temperature data taken from (Vanderwolf et al., 2017). “Mark2” refers to Markhamville Mine 2

Twilight zone air temperatures decreased as hibernaculum passage length increased (*F*_1_ = 185.5, *p* < .001), but the presence of water in hibernacula had a larger effect on temperature (*F*_2_ = 4,855.8, *p* < .001). Twilight zone temperatures were warmer at sites that contained flowing water (estimate = 6.87°C, *p* < .001) compared to sites with no water (estimate = 4.88°C, *p* < .001) or standing water only (estimate = 3.20°C, *p* < .001). Twilight zone air temperatures were colder than water temperatures during winter at sites with water flowing out of the hibernaculum entrance, such as Dorchester Mine and Kitts Cave, while we observed the opposite pattern at sites with water flowing into entrances, such as Harbells Cave (Figure 2). The R^2^ for fixed effects was 0.12 compared to 0.67 for the full model, indicating that year and month explain more twilight zone temperature variation than hibernaculum length and the presence of water.

Dark zone air temperatures increased as hibernaculum passage length increased (*F*_1_ = 7,092.3, *p* < .001) and were also affected by the presence of water (*F*_2_ = 6,808.0, *p* < .001). Dark zone temperatures were warmer for sites that contained flowing water (estimate = 6.24°C, *p* < .001) compared to sites with no water (estimate = 4.85°C, *p* < .001) or standing water only (estimate = 3.65°C, *p* < .001). The *R* ^2^ for fixed effects was 0.12 compared to 0.56 for the full model, indicating that year and month explain more temperature variation than passage length and the presence of water. A Wilcox test indicated that air temperature was not different from water temperature in the dark zone (W = 4,399, *p* = .203).

**FIGURE 3:**
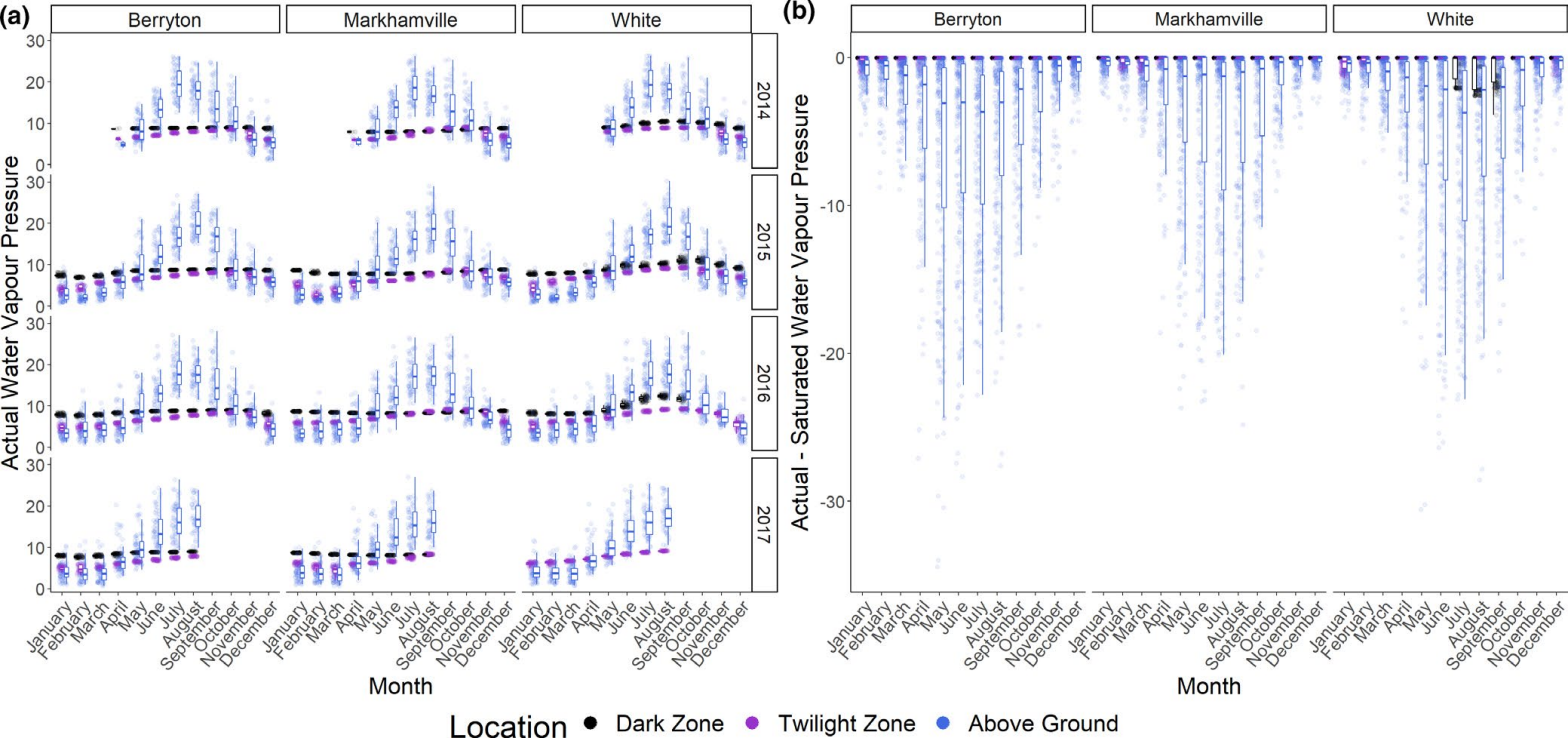
(a) Actual water vapor pressure as measured by HOBOs in two caves and a mine in New Brunswick 2014–2017. (b) Actual, minus saturated, water vapor pressure indicating when air is undersaturated

**FIGURE 4:**
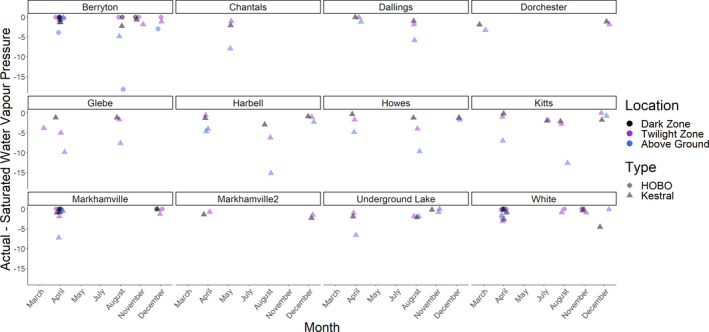
Actual, minus saturated, water vapor pressure indicating when air is undersaturated in New Brunswick caves and mines. Relative humidity and temperature measurements taken by Kestral weather meters during site visits. HOBO measurements taken the same day as Kestral measurements are indicated when available

Actual water vapor pressure varied with month (*F*_11_ = 2,672.2, *p* < .001), location within sites (*F*_2_ = 2,239.5, *p* < .001), year (*F*_3_ = 669.8, *p* < .001), and site (*F*_2_ = 336.7, *p* < .001; Figure 3). There was an interaction between month and location (*F*_22_ = 1,144.7, *p* < .001), site and location (*F*_22_ = 53.8, *p* < .001), and year and location (*F*_6_ = 2.3, *p* = .033). Actual water vapor pressure did not vary among months, sites, or years in the dark zone, while the pressure was higher in summer versus winter outside hibernacula and higher in early fall versus winter in twilight zones (Figure 3). Overall, the dark zone had a higher actual water vapor pressure (estimate = 8.01 hPa, *p* < .001) than either the twilight zone (estimate = 4.98 hPa, *p* < .001) or above ground (estimate = 3.52 hPa, *p* < .001). The dark zone and twilight zone were rarely undersaturated, unlike above ground (Figure 3). Actual water vapor pressure as measured by the Kestral weather meter was often lower than measurements recorded by HOBOs taken the same day (Figure 4). Likely this was because Kestral measurements were taken on the floor of hibernacula while HOBO measurements were taken near the ceiling. Kestral data indicate the air in the dark zone was close to saturation in all sites during winter.

Air temperature measurements taken by iButtons and HOBOs at the same locations within the same hibernacula at the same time were significantly different (W = 262,348,267, *p* < .001), with a 0.13°C ± 0.07 difference in the dark zone, 0.38°C ± 0.14 in the twilight zone, and 1.39°C ± 0.49 above ground. HOBO measurements were higher than iButton measurements at above ground locations (Table 7). Temperatures taken by paired iButtons (in the same box) differed from each other by 0.40°C ± 0.81 (V = 516,276,058, *p* < .001; *n* = 43,557 paired measurements).

**TABLE 7:** Mean temperature ± standard deviation (°C) taken by iButton and HOBO loggers in two caves and a mine in New Brunswick 2014–2017

Site	Dark zone		Twilight zone		Outside	
	iButton	HOBO	iButton	HOBO	iButton	HOBO
Berryton cave	4.88 ± 0.73	4.67 ± 0.93	0.57 ± 3.15	1.08 ± 2.82	6.38 ± 10.65	8.31 ± 10.85
Markhamville mine	4.43 ± 0.60	4.36 ± 0.57	0.99 ± 3.54	1.38 ± 3.26	5.55 ± 10.90	6.52 ± 10.77
White cave	6.63 ± 2.11	6.51 ± 2.09	3.33 ± 1.84	3.09 ± 2.40	6.63 ± 10.89	7.90 ± 10.77

More bats were counted in hibernacula of greater passage length (*F*_1_ = 4.48, *p* = .039) and fewer bats were counted toward the end of the study as WNS progressed (*F*_1_ = 3.49, *p* = .068). However, the number of bats did not vary with the presence of water bodies at sites (*F*_2_ = 0.54, *p* = .588), mean winter air temperature in the dark zone (*F*_1_ < 0.00, *p* = .990), standard deviation of winter temperatures in the dark zone (*F*_1_ = 0.33, *p* = .565), maximum winter temperature in the dark zone (*F*_1_ = 0.08, *p* = .775), or minimum winter temperature in the dark zone (*F*_1_ = 1.14, *p* = .291). The best model only included hibernacula passage length and year.

## 4 DISCUSSION

All known hibernacula in New Brunswick were *Pd*‐positive with WNS‐positive bats by winter 2013. A 99% decrease in the overwintering bat population in New Brunswick was observed between 2011 and 2015. We did not observe *P*. *subflavus* during surveys 2013–2015 and the species appears to be extirpated from these sites. As reported for hibernacula in the eastern USA (Wilder et al., 2011), WNS first appeared in New Brunswick among the largest known concentration of overwintering bats. Wilder et al. (2011) also note that those hibernacula with the highest proportions of *M*. *lucifugus* are more likely to exhibit high WNS mortality and most of our sites were dominated by that species. Bat species composition is uniform among New Brunswick hibernacula (two common species, one rare), and all three species exhibit high WNS‐associated mortality in the USA, particularly *M. septentrionalis* (Frick et al., 2017). Bat species that show resistance or tolerance to WNS, such as *E*. *fuscus* (Frank et al., 2014; Frick et al., 2017; Turner et al., 2011), were not present in our study sites, so it is unlikely bat species composition influenced the spread of *Pd* among New Brunswick hibernacula.

Our observations on the first occurrence of visible *Pd* growth on hibernating bats in New Brunswick are consistent with previous findings with respect to peak infection, although bats in Europe first exhibit visible fungal growth later in the hibernation season than in North America. In Europe, bats with visible *Pd* growth are first seen in January, with the number of cases slowly increasing into February, peaking in March (18%–55% of surveyed bats with visible fungal growth), and then dropping in April as bats emerge from hibernation (Puechmaille et al., 2011). In the USA, Lorch et al. (2011) detected WNS lesions in late September, but major mortality was not observed until the end of January, with peak mortality in March.

The pronounced drop in the overwintering bat population in New Brunswick mirrors bat mortality rates observed in the eastern USA (Powers et al., 2015; Langwig, Frick, et al., 2015; Langwig, Hoyt, et al., 2015; Turner et al., 2011). Turner et al., (2011), Langwig, Frick, et al. (2015) and Langwig, Hoyt, et al. (2015) noted significant variation among sites between the time of first WNS detection and the occurrence of mass mortality. In New Brunswick, three of our study sites (two mines and one cave), had delays of 10–12 months in bat population decreases following the first observation of *Pd* growing on bats, while the remaining five sites (one mine, four caves) had no delay. However, the lack of visible *Pd* growth on bats does not equate to the absence of WNS (Janicki et al., 2015). In a previous study using culture‐dependent methods, we detected *Pd* on 1/10 *Myotis* spp. in Howes Cave 24 February 2012 and on 3/11 *Myotis* spp. in Harbells Cave 28 Feb 2012, but visible *Pd* growth on bats at these sites was not observed until 27 March 2012 and 5 April 2013, respectively (Vanderwolf et al., 2016b). Therefore, the apparent lack of delay in some sites may be due to our inability to visually observe *Pd* infection.

The spike in bat numbers counted in Berryton Cave March 2011 and in Glebe Mine December 2012 was due to the movement of bats to roosting areas near cave entrances, facilitating counting. The movement of bats to hibernacula entrances is a behavioral change associated with WNS that has also resulted in an initial bat count increase at various hibernacula in the eastern USA (Powers et al., 2015; Turner et al., 2011). This behavior was not observed at any other New Brunswick hibernacula pre‐ or post‐WNS. Multiple areas of Berryton Cave are inaccessible to humans and presumably large numbers of bats roosted there prior to the onset of WNS. Unlike *Myotis* spp., we never observed *P*. *subflavus* roosting near hibernacula entrances with the onset of WNS. Our bat counts generally decreased from early hibernation to late hibernation, as found in the USA (Langwig et al., 2020). This may indicate mortality followed by predation (McAlpine et al., 2011) or the movement of bats to unknown hibernacula, although midwinter flights by *Myotis* spp. are considered rare (Davis & Hitchcock, 1965).

Bats in New Brunswick appear to have survived multiple years after the arrival of WNS, despite an overall major population decrease due to WNS and the continued presence of viable *Pd* on cave walls at hibernation sites (Vanderwolf et al., 2016a). However, because we did not mark bats individually, we cannot reject the possibility that some of these “survivors” are migrants from unaffected areas, but this seems unlikely because all adjacent provinces and states were WNS‐positive by winter 2013. Also, some surviving bats showed signs of previous exposure to *Pd*, with scarred wing membranes and truncated ear tips that may be the result of healed *Pd* damage. Surviving bats congregated in White Cave with little apparent change in roosting habits relative to pre‐WNS populations (roosting singly in the dark zone). It is unknown why White Cave has a greater proportion of survivors (38.4%–50% of surveyed bat population, fall 2013–winter 2015) than other sites in the province. The microclimate, habitat type, and physical features of White Cave are similar to other known hibernacula in the region and do not appear uniquely favorable for successful hibernation. Other studies have documented multiyear survival of bats post‐WNS, together with evidence of reproduction in the USA (Dobony & Johnson, 2018; Frick et al., 2017; Reichard et al., 2014). Additional monitoring will be necessary to ascertain if long‐term survival and reproduction is occurring among bat species that overwinter in New Brunswick post‐WNS.

It has been suggested that bats in Europe do not experience significant mortality from WNS because they form smaller hibernation groups (only rarely >1,000 individuals) than bats in eastern North America, and roost in small clusters or individually which may reduce pathogen transmission (Wibbelt et al., 2010). However, European and North American bats that roost singly in the winter (e.g., *P. subflavus* in North America) have also been confirmed WNS‐positive (Zukal et al., 2014), and European bats can develop lesions characteristic of WNS without mortality (Bandouchova et al., 2015). This suggests that clustering behavior does not explain the differences in mortality between European and North American bat populations. Although Langwig et al. (2012) and Wilcox et al. (2014) noted that bats cluster to a lesser degree as WNS infection progresses, most New Brunswick hibernacula already had small colonies of bats (<300) roosting singly or in small clusters (<100 individuals) pre‐WNS. Despite this pattern, all New Brunswick hibernacula ultimately experienced major population declines due to WNS. Dallings Cave, in which 0–6 hibernating bats roosted singly every winter, had WNS‐infected bats and associated mortality within 12 months of the first WNS occurrence in the province. Because viable *Pd* is readily cultured from hibernacula walls and cave sediments (Lorch et al., 2013; Puechmaille et al., 2011; Vanderwolf et al., 2016; Zhang et al., 2014), bats can likely acquire *Pd* from roosting substrates, as well as other bats (Langwig, Frick, et al., 2015; Langwig, Hoyt, et al., 2015). In the eastern USA, some bat species have high *Pd*‐loads, even in caves with <5 hibernating individuals roosting singly (Frick et al., 2017). This suggests that roosting individually offers no protection from *Pd* transmission once *Pd* is established in a hibernaculum due to environmental reservoirs and cryptic connections among bats (Hoyt et al., 2018).

Bats in our study area did not appear to select hibernacula based on temperature, and bats in all sites experienced high WNS‐associated mortality. However dark zone temperatures and water vapor pressure did not differ among our sites, likely because they were all located within a relatively small geographic area. However, bats may be selecting microclimates within sites that differ from our logger measurements. Bat species found in our sites can successfully hibernate in a wide range of temperatures (−4 to 17.8°C), although *P. subflavus* generally favor warmer temperatures than *M. lucifugus* or *M. septentrionalis* (Meierhofer et al., 2019; Webb et al., 1996). Wilder et al., (2011) suggest that bats in mines are less affected by WNS than in caves, possibly due to differing microclimates. Dark zone temperatures did not significantly differ between caves and mines in New Brunswick, nor was there a difference in bat mortality rates. Langwig et al. (2012) found that *M. lucifugus* population declines were higher in hibernacula with higher temperatures, which Johnson et al. (2014) and Grieneisen et al. (2015) also found in laboratory experiments. Conversely, Flory et al. (2012) found that WNS‐related mortality was higher in hibernacula with lower temperatures.

As found by previous studies, the presence of flowing water and passage length influenced hibernacula temperatures at our study sites (Perry, 2013), especially at the entrance. Hibernacula with longer passages are buffered from outside temperature fluctuations. Also, relatively warm water from the dark zone flowing toward the entrance may warm air temperatures in the twilight zone during winter. Significant differences in temperature data between paired iButtons and iButtons and HOBOs in the same locations are likely due to the large sample sizes. The dark zone and twilight zone differences between iButton and HOBO measurements and between paired iButtons were within the error range of iButtons (±1°C) and HOBOs (±0.45°C). Although iButtons and HOBOs were attached to the same tree outside hibernacula, HOBOs were always placed above iButtons, which may partially explain why HOBOs measured higher temperatures than iButtons above ground. Additionally, above ground the HOBO sensor was open to the air while iButtons were placed in a box, which may have influenced temperature readings (Terando et al., 2017).

Caves in the southeastern USA have experienced lower WNS‐related mortality compared to the northeast, possibly due to shorter hibernation periods and increased winter insect availability (Bernard & McCracken, 2017; Flory et al., 2012). Winter flights by bats (exiting hibernacula) are common in the southeastern USA (Bernard & McCracken, 2017). The increased fungal growth associated with warmer temperatures may influence disease outcomes less than the length of the hibernation period and food availability. In Canada, the duration of cold weather, and the subsequent length of hibernation, is even greater than in the northeastern USA (Norquay & Willis, 2014). Although optimal growth temperatures for *Pd* are 12.5–15.8°C in vitro (Verant et al., 2012), optimal conditions may differ for in vivo growth. We observed visible *Pd* growth on hibernating bats in New Brunswick during early winter surveys (mid‐November), even though hibernacula temperatures were cold (~4–5°C). This suggests that cold hibernacula temperatures do not slow the growth of *Pd* sufficiently to delay the onset of WNS and associated mortality over the winter. The province of Quebec, adjacent to New Brunswick, has experienced similar overwintering bat population declines as in New Brunswick (95% decline; Équipe de Rétablissement des Chauves‐souris du Québec, 2019). As WNS continues to spread west across continental North America, the severity of WNS‐related mortality may be greater at northern latitudes, where bat hibernation periods are longer relative to more southerly sites with shorter winters, despite apparent suboptimal temperatures for *Pd* growth.

## CONFLICT OF INTEREST

The authors declare no competing interests.

## AUTHOR CONTRIBUTION

**Karen J Vanderwolf:** Conceptualization (equal); Data curation (lead); Formal analysis (lead); Funding acquisition (supporting); Investigation (equal); Methodology (equal); Project administration (equal); Resources (equal); Software (lead); Visualization (lead); Writing‐original draft (lead); Writing‐review & editing (lead). **Donald F McAlpine:** Conceptualization (equal); Funding acquisition (lead); Investigation (equal); Methodology (equal); Project administration (equal); Resources (equal); Supervision (lead); Writing‐review & editing (supporting).

## Data Availability

Raw iButton, HOBO, Kestrel, and water temperature data is available in (Vanderwolf & McAlpine, 2020). All other data is included in the manuscript.
